# Tracking the Dynamic Functional Network Interactions During Goal-Directed Auditory Tasks by Brain State Clustering

**DOI:** 10.3389/fnins.2019.01220

**Published:** 2019-11-15

**Authors:** Gaoyan Zhang, Yuexuan Li, Jinliang Zhang

**Affiliations:** College of Intelligence and Computing, Tianjin Key Laboratory of Cognitive Computing and Application, Tianjin University, Tianjin, China

**Keywords:** dynamic network interaction, functional MRI, goal-directed auditory tasks, independent component analysis, brain state clustering

## Abstract

Both perceiving and processing external sound stimuli as well as actively maintaining and updating relevant information (i.e., working memory) are critical for communication and problem solving in everyday acoustic environments. The translation of sensory information into perceptual decisions for goal-directed tasks hinges on dynamic changes in neural activity. However, the underlying brain network dynamics involved in this process are not well specified. In this study, we collected functional MRI data of participants engaging in auditory discrimination and auditory working memory tasks. Independent component analysis (ICA) was performed to extract the brain networks involved and the sliding-window functional connectivity (FC) among networks was calculated. Next, a temporal clustering technique was used to identify the brain states underlying auditory processing. Our results identified seven networks configured into four brain states. The number of brain state transitions was negatively correlated with auditory discrimination performance, and the fractional dwell time of State 2-which included connectivity among the triple high-order cognitive networks and the auditory network (AN)-was positively correlated with working memory performance. A comparison of the two tasks showed significant differences in the connectivity of the frontoparietal, default mode, and sensorimotor networks (SMNs), which is consistent with previous studies of the modulation of task load on brain network interaction. In summary, the dynamic network analysis employed in this study allowed us to isolate moment-to-moment fluctuations in inter-network synchrony, find network configuration in each state, and identify the specific state that enables fast, effective performance during auditory processing. This information is important for understanding the key neural mechanisms underlying goal-directed auditory tasks.

## Introduction

Both perceiving and processing external sound stimuli as well as actively maintaining and updating relevant information (i.e., working memory) are critical for communication and problem solving in everyday acoustic environments (Huang et al., 2013). The translation of sensory information into perceptual decisions for goal-directed tasks hinges on dynamic changes in neural activity (Kopell et al., 2014). However, the dynamic changes in brain networks involved in this process are not well specified.

Functional connectivity (FC) is generally used to evaluate interactions in the brain, and it usually refers to the degree of co-variation between spatially distributed signals emanating from the brain. Interactions include FC among different brain regions that constitutes a local brain network and FC among different brain networks that constitute the large-scale brain network. Resting state fMRI measurements have shown that a brain network of auditory modality-specific areas in the temporal lobe participate in auditory processing (Damoiseaux et al., 2006). Task fMRI studies based on different cognitive loads have reported that distinct cortical networks were activated by auditory attention and working memory load (Huang et al., 2013), and FC between the supratemporal plane (STP) and inferior parietal lobule (IPL) in the auditory network (AN) was modulated when discriminating and actively maintaining different pitch-varying sounds (Hakkinen and Rinne, 2018). Another study on auditory word processing based on FC analyses demonstrated that auditory processing recruited the language network (LN), the dorsal attention network (DAN), and the default mode network (DMN). This study also found that intra-network connectivity was stronger in one language than in another (Jung et al., 2018).

Although previous studies have suggested that multiple brain networks are involved in processing auditory goal-directed tasks, it should be noted that these FC studies are commonly conducted based on the hypothesis that FC in the human brain is stable. Correspondingly, the network dynamics during the auditory process are unclear. Recent work has increasingly found that FC is dynamic and evolves in biologically meaningful ways at temporal scales ranging from years to seconds (Gonzalez-Castillo and Bandettini, 2018). At shorter temporal scales, FC patterns computed over tens of seconds contain sufficient information to determine the tasks in which subjects are actively engaged (Shirer et al., 2012; Gonzalez-Castillo et al., 2015). A study that used magnetoencephalographic signals to assess human listeners judging acoustic stimuli composed of carefully titrated clouds of tone sweeps, suggested that global network communication during perceptual decision-making was implemented in the human brain by large-scale couplings between beta-band neural oscillations (Alavash et al., 2017). However, how large-scale functional network interactions change dynamically in the temporal domain and how different cognitive loads modulate dynamic functional network connectivity (FNC) in auditory tasks is still unclear. Further investigation of these unsolved questions is important to improve our understanding of how these processes support goal-directed functioning in everyday acoustic environments.

The recent development of time-resolved analyses of functional neuroimaging data provide a unique opportunity to examine time-varying reconfigurations in global network structure (Shine et al., 2016). Many studies now use independent component analysis (ICA) to extract brain networks and assess dynamic changes in connectivity strength among networks to explore the neural mechanisms underlying development and brain disease (Faghiri et al., 2018). In this study, we used this method to track the dynamic changes in FNC during different auditory tasks. We also assessed the modulation of task load on FNC and its correlation with cognitive behaviors. We believe that this dynamic FNC analysis may reveal detailed information regarding brain dynamics during auditory goal-directed tasks.

## Materials and Methods

### Participants

Twenty college students (mean age: 22.5 years, age range: 20–24, 10 female, right handed) participated in this study. They all had normal hearing, with no history of neurological disorders.

### Experiments

The whole experiment included one auditory discrimination run and one auditory working memory run, with a total length of 402s for each run. Both runs started with an 8-s fixation, followed by eight 36-s sound blocks interleaved with eight 12-s resting blocks. The eight task block included four sound-source categories (two living categories of animal sounds and human sounds and two non-living sound-source categories of traffic sounds and tool sounds) intersected with two directions (left and right) (Engel et al., 2009). In each sound block, 12 sound samples (with same category and direction) were randomly presented, and each lasted for 2.5 s with an inter-sample-interval of 0.5 s. In the auditory discrimination task, participants were asked to judge whether the current sound samples were same as the first sound samples in that block (0-back). For the sound blocks in the auditory working memory task, a 2-back paradigm was used; here, participants were instructed to judge whether a current sound sample was same as the one given two samples before.

### Data Collection

Imaging data were acquired using a 3.0-T SIEMENS MRI scanner. An eight-channel head coil was used during scanning. Foam pads and earplugs were used for all participants to reduce head motion and scanner noise. To prevent visual input from distracting participants from the auditory task, eyeshades were worn by participants during testing. T2^∗^-weighted images were acquired using a gradient echo-planar imaging (EPI) sequence with the following parameters: repetition time (TR) = 2000 ms, echo time (TE) = 30 ms, voxel size = 3.1 × 3.1 × 4.0 mm^3^, matrix size = 64 × 64, slices = 33, slice thickness = 4 mm, slice gap = 0.6 mm. T1-weighted anatomical images were acquired using a three-dimensional magnetization-prepared rapid acquisition gradient echo (3D MPRAGE) sequence with the following parameters: TR = 1900 ms, TE = 2.52 ms, time of inversion (TI) = 1100 ms, voxel size = 1 × 1 × 1 mm^3^, matrix size = 256 × 256. Participants perceived auditory stimuli through the earphones of the VisuaStim Digital MRI Compatible fMRI system.

### Data Preprocessing

The DPABI toolbox^1^ was employed for data preprocessing. For each run, the first four images were removed to minimize magnetic saturation effect. Slice timing and head motion correction were performed for the remaining functional images. The translation and rotation parameters of head motion were less than 2 mm and 2°. We also calculated the framewise displacements using a method reported in a previous study (Jenkinson et al., 2002). The framewise displacements were 0.04 ± 0.01 and 0.04 ± 0.02 for the two runs, demonstrating the head motion across frames was controlled well. Next, structural T1-weighted images were co-registered to the mean functional image, and then normalized to Montreal Neurological Institute (MNI) space using a non-linear registration. EPI data were spatially normalized to MNI space with warping parameters estimated from coregistered, high-resolution T1 images, and voxel size was re-sampled as 3 × 3 × 3 mm^3^. The normalized data were then smoothed with a 6-mm full-width half-maximum Gaussian kernel to improve the signal-to-noise ratio. After that, experimental paradigm convolved with the canonical hemodynamic response function was used as a regressor in a general linear model to calculate the brain activation map in each task. Six head motion parameters and their derivatives was used as covariates. For the FC analysis, six head motion parameters and their derivatives, as well as the experimental paradigm convolved with the canonical hemodynamic response function, were regressed out of the smoothed fMRI time series. The residual was used for the task-state FC analysis to exclude the artificial correlation between networks induced by shared activations (Poldrack et al., 2011).

### Functional Network Extraction

**FIGURE 1:**
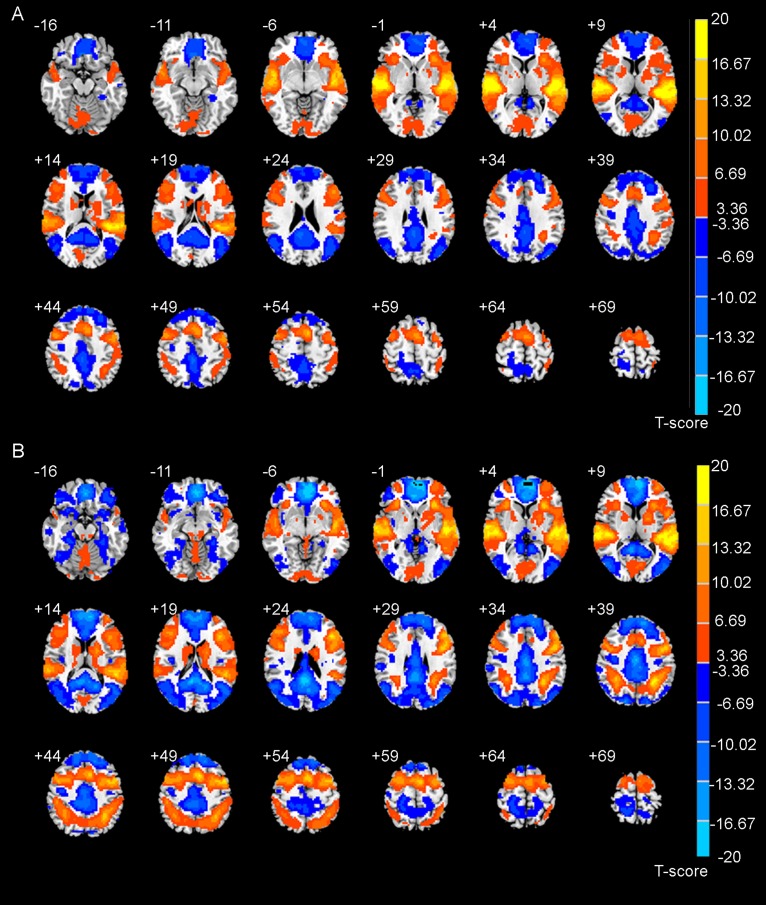
The brain activation maps during auditory discrimination task **(A)** and auditory working memory task **(B)** calculated by general linear model.

Functional brain network data were extracted using the group ICA method implemented by the GIFT toolbox^2^. Spatially independent component maps and their respective time series were extracted from the data using the following steps. For each subject, preprocessed data were first reduced to 27 components using principle component analysis. Next, individual data were appended along the time dimension and another principle component analysis was performed for group level dimension reduction, from which 18 components were retained. The number of components was estimated based on the minimum description length criterion. Once this had been performed, the infomax algorithm was applied for ICA; here, the algorithm was run 10 times to reduce the effect of subject order. The results were clustered via ICASSO^3^ and the most central solution was used to ensure stability. For all components, the stability index of ICA estimate-clusters was around 1, demonstrating that the result was stable even though the subject order was adjusted. Using the back-reconstruction approach, the spatial maps and time courses for each subject were extracted. After visually checking all components, those with a peak in white matter, ventricles, brain stem, or cerebellum, or those with a spatial map and time course dominated by high frequency fluctuations (likely due to motion or physiological effects) were removed. Fourteen components remained based on brain activation maps of the two tasks (Figure 1) in which significant activations were observed in auditory regions, visual regions, sensorimotor regions, cerebellar regions, frontoparietal regions, DMN regions and frontoinsular regions in salience network (SN; *p* < 0.001, corrected for false discovery rate). Previous studies about the brain networks involved into auditory cognitive tasks (Schneiders et al., 2012; Huang et al., 2013; Kumar et al., 2016) were also referred to. Finally, the 14 components were grouped into 7 functional brain networks.

### Dynamic FNC Calculation

**FIGURE 2:**
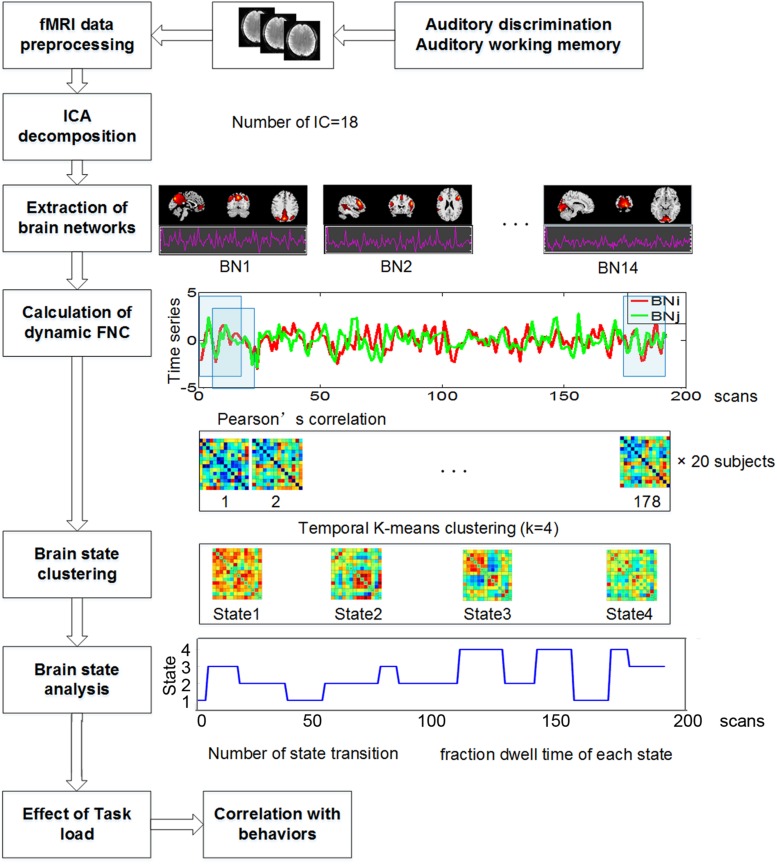
The whole data processing steps.

The whole data processing steps were illustrated in Figure 2. For time series data of the 14 selected components, we first performed linear detrending and low pass filtering (0.1 Hz). Next, a sliding-window approach was used to calculate dynamic FNC. A window size of 30s was selected according to previous studies, which suggested that 30s–60s of data can effectively capture dynamic information (Hutchison et al., 2013; Allen et al., 2014; Faghiri et al., 2018). A tapered window was created by convolving a Gaussian with a rectangular function. For each window, a full correlation matrix was calculated. The sliding step was 1 sample, resulting in a total of 178 dynamic FNC matrices.

### Brain State Clustering and State Analysis

To examine the reoccurring FNC patterns in the temporal process, we used k-means clustering on all sliding-window FNC matrixes of all subjects by Manhattan distance because L^1^ distance is more suitable for calculating similarity of high-dimensional data (Charu et al., 2001). A maximum iteration of 150 was used on the time-varying FNC matrices to cluster brain states. Different number of clusters was calculated from 2 to 10. Through dividing within- by between-cluster distances, four clusters was determined by the elbow criterion of the cluster validity index.

After obtained the four brain states, state transition was defined as the number of times a subject transitioned from one state to another. The time proportion of each subject stayed in each state within the whole task duration was defined as fractional dwell time in that state. Due to the non-normality of the two measures after Kolmogorov-Smirnov test (the number of state transitions: *p* = 0.026, and the fractional dwell time in four states: *p* = 0.034, 0.021, 0.117, and 0.200) with SPSS 22.0 software^4^, the number of state transitions and the fractional dwell time of each state were separately compared between the two tasks using permutation test. The permutation test was performed as follows. Mean inter-group difference of each measure was calculated firstly, and then all the values of this measure were randomly reassigned into the two groups for 10,000 times. If less than 5% of mean values of randomized inter-group differences were equal or larger than the mean value of original inter-group differences, the result was seemed as significant (*p* < 0.05). In addition, the spatial strength of each state was also compared between tasks using paired *t*-tests (*p* < 0.05, corrected for false discovery rate).

### Correlation Analysis of Brain State Measure With Behavior

For the auditory discrimination and auditory working memory tasks, dprime scores (Haatveit et al., 2010) were calculated separately to evaluate behavioral performance. Pearson correlations of dprime score with number of state transition and fractional dwell times were conducted to examine whether the dynamic brain network states were related to behavior. The framewise displacement of each subject was used as a covariate in the partial correlation analysis. The significance of the results was tested using fisher *t*-test (*p* < 0.05).

## Results

### Spatial Maps of Task-Related Functional Brain Networks

**FIGURE 3:**
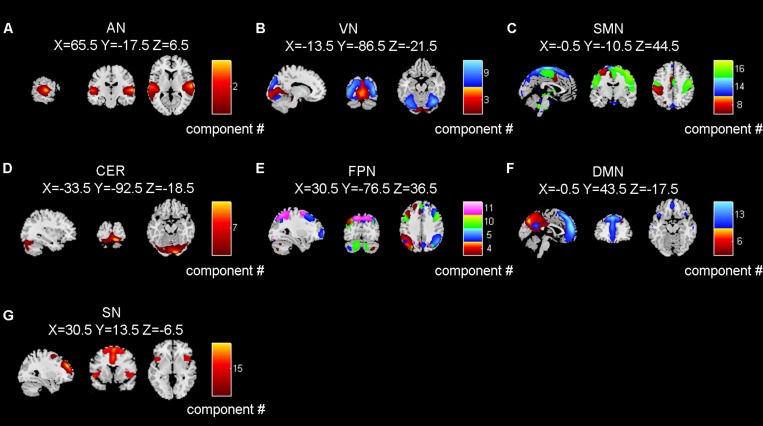
The seven brain networks extracted by independent component analysis. **(A–G)** refer to AN (auditory network), VN (visual network), SMN (sensorimotor network), CER (cerebellum network), FPN (frontoparietal network), DMN (default mode network), and SN (salience network), respectively. Number in color bar means component number. Brain networks in panels **(B,C,E,F)** were composed by multiple independent components.

After removing the components related to artifacts, we selected 14 task-related brain network components based on the spatial maps and frequency distribution as mentioned in the method section. The extracted 14 independent components were distributed in 7 functional networks, including the AN, the visual network (VN), the sensorimotor network (SMN), the cerebellar network (CER), the frontoparietal network (FPN), the DMN, and the SN (Figure 3). The spatial maps of functional networks were displayed using a threshold of *z*-score > 2.0 and multiple components within one functional network were displayed in a composite plot.

### Dynamic Functional Network Connectivity Patterns

**FIGURE 4:**
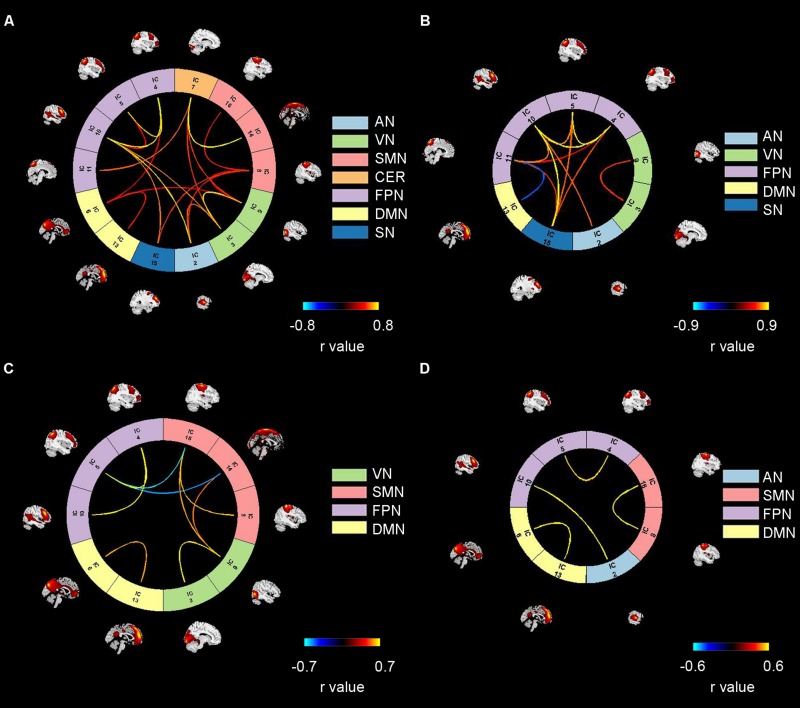
Four reoccurring brain states during auditory processing revealed by clustering analysis. Panels **(A–D)** refer to State 1, 2, 3, 4. Color bars refer to the connectivity strength. AN: auditory network; VN: visual network; SMN: sensorimotor network; CER: cerebellum network; FPN: frontoparietal network; DMN: default mode network; SN: salience network.

The dynamic interactions among the seven functional networks were evaluated using a sliding-window correlation analysis method on the corresponding time series. The 178 dynamic FNC matrices were clustered into 4 brain states. For better visualization, each state was represented by its centroid and is shown in Figure 4 using a threshold of absolute correlation value *r* > 0.5 (The original connectivity matrix is shown in Supplementary Figure S1).

As shown in Figure 4, State 1 consisted of all seven brain networks. In State2, strong connectivity was observed in three higher cognitive networks (FPN, DMN, SN) and two primary networks (AN and VN). In this state, we can see strong inter-network interactions among the three higher cognitive networks as well as between a higher cognitive network (FPN) and a primary network (AN). In contrast, strong FC in State 3 and State 4 involved two cognitive networks (DMN, FPN) and two primary networks (SMN, VN or AN). In State 3, we observed strong cross-network interactions between FPN and SMN as well as between SMN and VN. With respect to State 4, only one strong inter-network interaction was found between FPN and AN.

### Brain State Analysis Results

The average state transition times and fractional dwell time of each state for the two tasks are listed in Table 1. The number of state transitions were similar for both tasks (permutation test, *p* = 0.17). For fractional dwell time, it can be seen that nearly the same percentage of time was spent in the two tasks for States 3 and 4, while the fractional dwell times of State 1 and State 2 showed opposite trends in the auditory discrimination and auditory 2-back tasks. When comparing the fractional dwell times of each state between the two tasks, there was no significant differences (permutation test, *p* = 0.33, 0.20, 0.98, and 0.96, respectively for the four states).

**TABLE 1:** The average state transition times and fractional time in each state for the two tasks.

	**No. of state transition**	**Fractional dwell time in each state**			
		
		**State 1**	**State 2**	**State 3**	**State 4**
Auditory discrimination	10.15 ± 0.93	0.26 ± 0.05	0.15 ± 0.03	0.22 ± 0.05	0.37 ± 0.05
Auditory 2-back	11.7 ± 0.64	0.19 ± 0.04	0.21 ± 0.04	0.22 ± 0.03	0.37 ± 0.04

**FIGURE 5:**
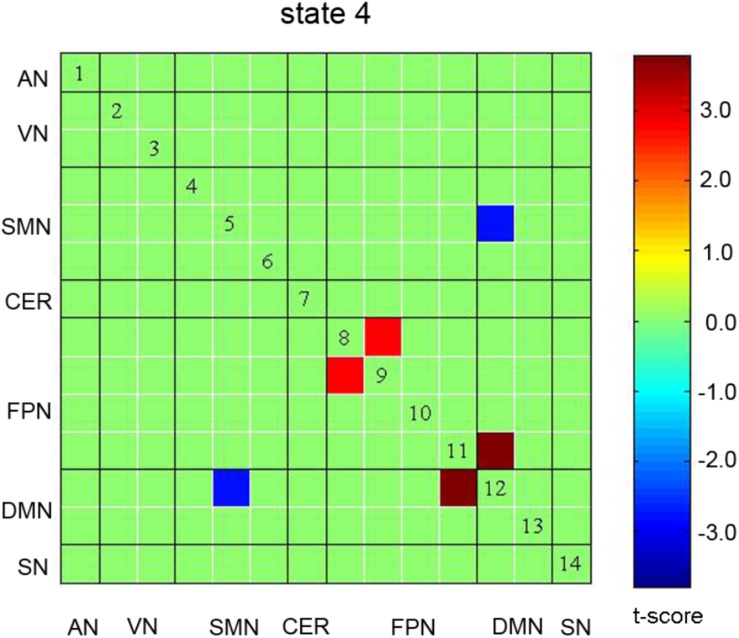
Significant differences in spatial pattern of State 4 in the comparison of auditory discrimination with auditory working memory tasks. No significant differences were observed in other States. AN: auditory network; VN: visual network; SMN: sensorimotor network; CER: cerebellum network; FPN: frontoparietal network; DMN: default mode network; SN: salience network.

The spatial pattern of each state was also compared between the auditory discrimination task and the auditory working memory task. Significant differences were observed for States 1, 3, and 4, but only differences in State 4 were retained after correcting for multiple comparisons (see Figure 5). In State 4, stronger negative connectivity between FPN and DMN and stronger positive connectivity between DMN and SMN were found for the working memory task, while stronger positive connectivity within FPN was found for the auditory discrimination task (*p* < 0.05, FDR corrected).

### Correlation Results of Brain State Measure With Behavior

**FIGURE 6:**
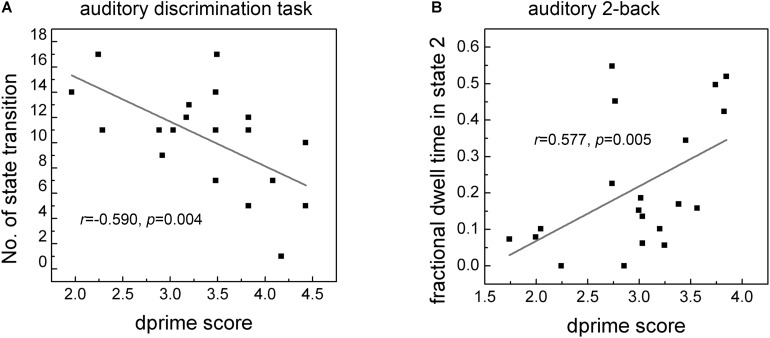
Correlation of dprime score with the number of state transition **(A)** and fractional dwell time in corresponding state 2 **(B)** in the two tasks. Only fractional dwell time in State 2 showed significant correlation with dprime score of auditory 2-back task.

Significant correlation of dprime score with the number of state transition and with fractional dwell time in different states for the two tasks were separately reported in Figure 6. It can be seen clearly that a negative correlation (*r* = −0.590, *p* = 0.004, Figure 6A) between dprime scores and the number of state transitions in the auditory discrimination task. For the auditory working memory task, there was a positive correlation (*r* = 0.577, *p* = 0.005, Figure 6B) between dprime scores and the fractional dwell time in State 2. No significant correlation was observed between dprime scores and the fractional dwell time in other States.

## Discussion

In this study, we used data-driven ICA method to extract functional brain networks, and then a temporal clustering analysis on the sliding-window FNC to reveal the time-variable FNC pattern (i.e., brain state) during two goal-directed auditory tasks. This approach allowed us to isolate moment-to-moment fluctuations in inter-network synchrony, which were related to behavioral variability during auditory discrimination and working memory tasks. The findings in this study also reveal the modulation of cognitive demands on the connectivity of time-variable functional networks. Altogether, this study provides a new perspective on time-sensitive shifts in brain network interactions, and this may help us understand the key neural mechanisms underlying goal-directed auditory tasks.

In this study, seven brain networks were found to be configured into four states. State 1 included all networks. States 2, 3, and 4 mainly included strong connectivity in FPN, DMN, AN, VN, and SMN, but the interaction patterns of these networks differed in each state, demonstrating that these states may contribute to different cognitive processing. The network configuration is consistent with previous auditory cognitive studies (Schneiders et al., 2012; Huang et al., 2013; Kumar et al., 2016). For example, using auditory near perception threshold (NT) paradigms, researchers observed that a stronger integration of the auditory network with the frontoparietal and other high-order cognitive networks was key for subsequent auditory performance (Leske et al., 2015). In another study, researchers investigated the brain system for actively maintaining sound memory over short periods of time (Kumar et al., 2016). Their results supported the hypothesis that a system maintained sound-specific representations in the auditory cortex by projecting from higher-order areas, including the hippocampus and frontal cortex. Another recent study documented that the activation of the auditory cortex and adjacent regions in the IPL were strongly modulated during active listening and depended on task requirements (Wikman and Rinne, 2018). In contrast to these studies, we investigated network interactions from a dynamic perspective and found that brain States 2 and 4 mainly showed a strong interaction between FPN and AN, suggesting that these two states may contribute to sound maintenance and active listening. State 3 contained strong interactions in both FPN-SMN and SMN-VN connectivity. The involvement of SMN, which is important in motor output (De Luca et al., 2005), implies that these interactions may contribute to cognitive decision and button-press. Further studies with high temporal resolution technique are needed to verify the inferences.

Moreover, a comparison between the auditory working memory and discrimination task datasets showed significant differences in State 4, demonstrating that State 4 is an indicator of cognitive load. The load-related increases in connectivity among cognitive (FPN and DMN) and SMNs are coincident with the finding of increased task-driven connectivity between the frontoparietal, dorsal attention, and sensory networks by a previous study (Shine et al., 2016). Moreover, the increased negative correlation of FPN-DMN connectivity in working memory task is consistent with a previous finding (Schneiders et al., 2012), and further reveal the cognitive resources demanded for sound maintenance in this state. These results also suggest that global integration may have facilitated communication during the more challenging working memory task.

Interestingly, by using dynamic network analysis in this study, shifts among brain networks can be measured and the brain-behavior relationship showed that the number of brain state transitions was negatively correlated with auditory discrimination performance, meaning that fewer state transitions contribute to better behavioral performance, but this is not the case for the auditory working memory task. In this study, we also found that the fractional dwell time in State 2 was positively correlated with auditory working memory behavior. In State 2, the triple networks (i.e., FPN, DMN, and SN) and the typical FPN-DMN anticorrelation were most prominent. The triple networks have been suggested as the most crucial components of a unified network model and are thought to be extensively involved in diverse cognitive functions (Menon, 2011; Li et al., 2018). A strong competitive relationship between FPN and DMN was previously reported to be significantly correlated with working memory behavior (Hampson et al., 2010) and may represent a cerebral mechanism that switches mental focus between internal channels (supported by DMN) and external, attention-demanding events (Hampson et al., 2010). Moreover, the connectivity between the high-order cognitive network (FPN) and the primary network (AN) was also found to be significant in this state. Taken in the light of these previous studies, our findings suggest that State 2 probably contributes to top-down attention switching, cognitive processing, and behavioral modulation processing (Zhang et al., 2015; Le Merre et al., 2018), all of which are necessary for good performance. Therefore, more time spent in this state should correspond to better auditory memory performance.

## Conclusion

The human brain network traverses segregated and integrated states over time (Shine et al., 2016). The dynamic FNC analysis used in this study can help identify network configurations of each state, as well as the specific states that enable fast, effective performance on goal-directed auditory tasks. Other approaches are also used in investigating dynamic brain connectivity analysis (Calhoun and Adali, 2016) and a dynamic approach can also be found with a stable non-dynamic model. Future studies can further explore the effectiveness of different methods. In summary, building on the results of previous auditory cognitive studies, the dynamic functional network analysis in this study enrich our understanding of the neural mechanisms underlying auditory discrimination and working memory.

## Data Availability Statement

The datasets generated for this study are available on request to the corresponding author.

## Ethics Statement

This study was approved by the Research Ethics Committee of Tianjin Key Lab of Cognitive Computing and Application, Tianjin University. All procedures followed were in accordance with the ethical standards of the responsible committee on human experimentation (institutional and national) and with the Helsinki Declaration of 1975, and the applicable revisions at the time of the investigation. Informed consent was obtained from all subjects for being included in the study.

## Author Contributions

GZ designed the experiment, and wrote and revised the manuscript. JZ collected the data. GZ and YL contributed to the data analysis.

## Conflict of Interest

The authors declare that the research was conducted in the absence of any commercial or financial relationships that could be construed as a potential conflict of interest.

## Footnotes

1 http://www.rfmri.org/dpabi

2 http://mialab.mrn.org/software/gift

3 http://research.ics.aalto.fi/ica/icasso/

4 https://www.ibm.com/analytics/spss-statistics-software
