# Supplementary material for: Tracking the Dynamic Functional Network Interactions During Goal-Directed Auditory Tasks by Brain State Clustering
Source: Front Neurosci. 2019 Nov 15;13:1220. doi: 10.3389/fnins.2019.01220 (PMC6872968; doi:10.3389/fnins.2019.01220)
Supplement: Supplementary file 1 [file Data_Sheet_1.PDF]

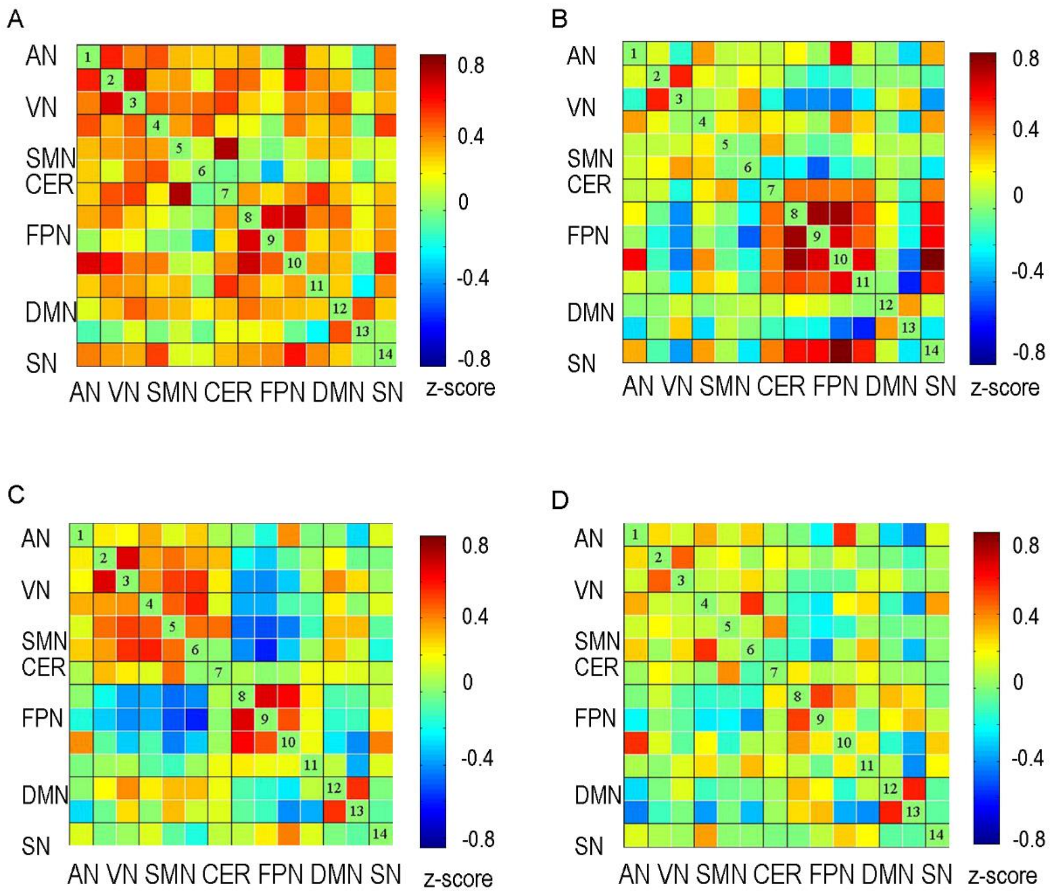

Fig. S1. The four brain state connectivity matrix extracted by clustering analysis. AN: auditory network; VN: visual network; SMN: sensorimotor network; CER: cerebellum network; FPN: frontoparietal network; DMN: default mode network; SN: salience network.
